# A novel fungus concentration-dependent rat model for acute invasive fungal rhinosinusitis: an experimental study

**DOI:** 10.1186/s12879-014-0713-y

**Published:** 2014-12-20

**Authors:** Yuyan Yan, Zuotao Zhao, Hongfei Wan, Ruochen Wu, Jugao Fang, Honggang Liu

**Affiliations:** Department of Pathology, Affiliated Beijing Tongren Hospital, Capital Medical University, Beijing, 100730 China; Department of Dermatology, First Hospital, Peking University, Beijing, 100034 China; Department of ENT, Affiliated Beijing Tongren Hospital, Capital Medical University, Beijing, 100730 China

**Keywords:** Acute invasive fungal rhinosinusitis, Animal model, Aspergillus fumigatus, Mast cells

## Abstract

**Background:**

Acute invasive fungal rhinosinusitis is a lethal infectious process afflicting immunocompromised individuals. Knowledge about this disease is still limited due to the scarcity of animal models designed to study the pathogenesis of this infection. Mast cells are tissue-resident immune cells that participate in a variety of allergic and inflammatory conditions. Limited attention has been given to the role of mast cells in acute invasive fungal rhinosinusitis. Therefore, the objectives of this study were to create a rat model of acute invasive fungal rhinosinusitis based on analyzing the impact of different fungal concentrations on establishing infection, and to observe the changes of mast cells in rats with this disease.

**Methods:**

Sprague–Dawley rats were divided randomly into four groups, three of which were experimental and received different concentrations of *Aspergillus fumigatus* inoculations, and one was a control group (D). The inoculated *Aspergillus fumigatus* concentrations were 5 × 10^7^ conidia/ml in group A, 10^7^ conidia/ml in group B, and 10^6^ conidia/ml in group C. Before fungal inoculation, rats were immunosuppressed using cyclophosphamide and cortisone acetate, and had Merocel sponges inserted into the right nares. Hematology and histopathology investigations were then performed.

**Results:**

An acute invasive fungal rhinosinusitis rat model was established successfully with an incidence rate of 90% in group A, 50% in group B and 10% in group C. *Aspergillus fumigatus* invasion was observed in 20% of the lungs in group A, but was not seen in the remaining groups. In addition, no fungi invaded the orbital tissue, brains, livers, spleens or kidneys of any rat. Compared with the control set, the total number of mast cells in the experimental groups was not significantly increased, but mast cell degranulation, on the other hand, was only found in infected nasal cavities.

**Conclusions:**

This investigation illustrates that various fungal concentrations have different effects on the incidence of acute invasive fungal rhinosinusitis, and it also demonstrates the feasibility of using this model to study the process of fungal rhinosinusoidal invasion. In addition, the results suggest that mast cells may play a role in the protection of sinuses against acute *Aspergillus fumigatus* infection and in the clearance of established hyphal masses.

**Electronic supplementary material:**

The online version of this article (doi:10.1186/s12879-014-0713-y) contains supplementary material, which is available to authorized users.

## Background

Acute invasive fungal rhinosinusitis (AIFR) is an aggressive fungal infection with high mortality rates (50%–80%) in immunocompromised patients [1],[2]. It is characterized by fungal invasion into the mucosa and submucosal structures of the nasal cavity or paranasal sinuses with frequent extension into adjacent structures, including the nasal soft tissue, vasculature, orbit, and cranium [3]-[5]. Hematological–oncological malignancies, aplastic anemia, uncontrolled diabetes mellitus, and acquired immune deficiency syndrome (AIDS) are the most common predisposing diseases for AIFR [6]-[9]. In addition, long-term steroid treatment and the utilization of chemotherapy agents and immunosuppressants, which are used after bone marrow and solid organ transplantations, are important risk factors [10]-[12].

At present, animal models designed for the study of sinusitis are limited to bacteria [13]-[17]. For fungal infections, most studies focus on pulmonary aspergillosis and noninvasive fungal sinusitis [18]-[23]. Only two papers presenting an AIFR animal model are currently published [5],[24]. Due to the lack of an established AIFR animal model that closely mimics the pathophysiology of infection in addition to providing ease of operation, most AIFR research has been limited to clinical observations [2],[3],[12],[25].

Mast cells (MCs) are leukocytes that are derived from haematopoietic progenitor cells. They are long-lived and reside in most tissues of the body, particularly in locations that are in close contact with the external environment, such as skin, airways, and intestines, where they can initiate and enhance early responses to environmental threats, including pathogens [26]. MCs are preferentially located in the vicinity of blood vessels, nerves, and lymphatic vessels, where they can regulate vascular permeability and instigate effector cell recruitment by releasing preformed as well as de novo synthesized mediators [27]. MCs are also important effector cells in allergic diseases and protective immune responses against pathogens, with roles in both innate and adaptive immunity, including the direct killing of organisms [28]-[32]. Because the majority of MC research focuses on bacteria, the role of MCs in the pathogenesis of fungal infections is poorly understood. A single in vitro study found that *Aspergillus fumigatus* hyphae induced degranulation of MCs via an IgE independent mechanism [33]. In normal subjects and those with non-allergic rhinitis, there are few MCs in the epithelial compartment of the nasal mucosa. Studies have found that the number of MCs increases in allergic rhinitis and nasal polyps [34]-[36]. Currently, it is still unclear what types of changes are occurring in the MC population in AIFR.

The focus of our study was to create a novel AIFR rat model by analyzing the impact of different fungal concentrations on AIFR establishment, and then to use this model to explore the role that MCs may play in the disease process. In order to develop a novel animal model of AIFR that is stable and clinically relevant, we tested numerous immunosuppression regimens and various concentrations of fungal spores. The intranasal infection model reported here illustrates that various concentrations of fungi have different effects on AIFR incidence. With the appropriate concentration of fungi, an AIFR model that is stable and clinically relevant can be established in the immunosuppressed rat, and this model can be used to study immune factors, such as MCs, related to AIFR.

## Methods

### Rats and groups

Forty female Sprague–Dawley (SD) rats aged 6–8 weeks and weighing 230 ± 10 g were used. They were housed in pathogen-free conditions in the Animal Laboratory of the First Affiliated Hospital of General Hospital of Chinese People's Liberation Army (PLA) of Beijing, and the rats were given food and water at all times. All procedures and animal care were conducted in accordance with guidelines set forth by the Institutional Animal Care & Use Committee (IACUC) of First Hospital Affiliated to PLA General Hospital and adhere to Reporting In Vivo Experiments (ARRIVE) guidelines (http://www.nc3rs.org.uk/page.asp?id=1357). The animal study was approved by the Institutional Animal Care & Use Committee (IACUC) of First Hospital Affiliated to PLA General Hospital.

Rats were randomly divided into four groups of 10. Table 1 shows the interventions for each group. The model was established in three consecutive steps: 1) Cyclophosphamide (CPA) and cortisone acetate (CA) were administered, 2) Unilateral nasal cavities were obstructed with Merocel sponges (Medtronic Xomed, Jacksonville, FL), and 3) Nasal cavities were inoculated with *A. fumigatus*. The different experimental groups were inoculated with various concentrations of *A. fumigatus*: group A received 5 × 10^7^ conidia/ml, group B 1 × 10^7^ conidia/ml, and group C 1 × 10^6^ conidia/ml. Group D was the control group and received no treatment.

**Table 1:** **Groups and interventions**

Group	n	Intervention
A	10	Administration of immune inhibitors with nasal obstruction and fungal inoculation of 5 × 10^7^ conidia/ml concentration
B	10	Administration of immune inhibitors with nasal obstruction and fungal inoculation of 10^7^ conidia/ml concentration
C	10	Administration of immune inhibitors with nasal obstruction and fungal inoculation of 10^6^ conidia/ml concentration
D	10	No treatment (negative control)

n: Number of rats in each group.

### Immunosuppression

Based on many preliminary experiments, we chose cyclophosphamide (Sigma, America) [37],[38] and cortisone acetate suspension (Sigma, America) [39]-[41] as immune inhibitors to establish immunosuppressed rats before nasal obstruction and fungal inoculation.

Table 2 shows the doses, timing and methods of cyclophosphamide and cortisone acetate delivery. Five days before the first administration of *A. fumigatus* (D-5), rats in groups A, B and C were given an intraperitoneal injection (ip) of CPA at a dose of 75 mg/kg and a subcutaneous injection (sc) of CA at a dose of 80 mg/kg. One day before the first administration of *A. fumigatus* (D-1), rats in groups A through C were given a CPA ip at a dose of 60 mg/kg and a CA sc at a dose of 80 mg/kg. The last injections of CPA at a dose of 50 mg/kg and CA at a dose of 80 mg/kg were given two days after the first administration of *A. fumigatus* (D + 2).

**Table 2:** **Rat immunosuppressant doses (mg/kg)**

Immunosuppressant	D-5	D-1	D + 2
CPA (ip)	75	60	50
CA (sc)	80	80	80

CPA: Cyclophosphamide; CA: Cortisone acetate; D-5: Five days before the first administration of *A. fumigatus* spores; D-1: One day before the first administration of *A. fumigatus* spores; D + 2: Two days after the first administration of *A. fumigatus* spores; ip: Intraperitoneal injection; sc: Subcutaneous injection.

For the neutrophil count analysis, whole blood samples (0.1 ml) were collected from the angular veins of all rats on D-5, the first administration day of *A. fumigatus* spores (D0), and the fourth day after the first administration of *A. fumigatus* (D + 4). The blood counts were analyzed with SYSMEX XS-820 (Japan) in the Laboratory of Beijing Tongren Hospital.

### Fungi inoculum

*A. fumigatus* strain (AF9732 wild strain from the Fungi and Fungal Disease Research Center of Peking University) was grown on potato dextrose agar (PDA) plates at 37°C for 5 days. Conidia were harvested by washing the plates with sterile 0.2% Tween 20, followed by centrifugation and filtration of the suspension through eight layers of sterile gauze to remove hyphae. The conidia were subsequently diluted and counted with a hemocytometer to obtain 5 × 10^7^, 1 × 10^7^ and 1 × 10^6^ conidia/ml suspensions.

### Nasal obstruction and intranasal inoculation of *A. Fumigatus*conidia

**Figure 1:**
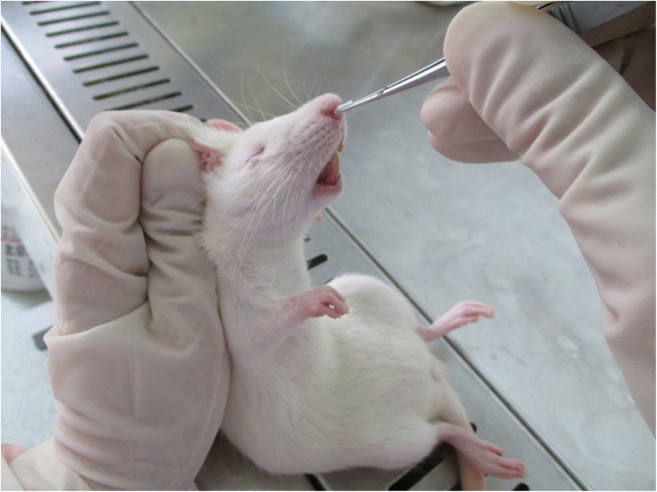
**Insertion of Merocel sponge into right nasal cavity of rat.** Merocel sponges were gently inserted 1.5 cm deep into the rats’ right nasal cavities.

**Figure 2:**
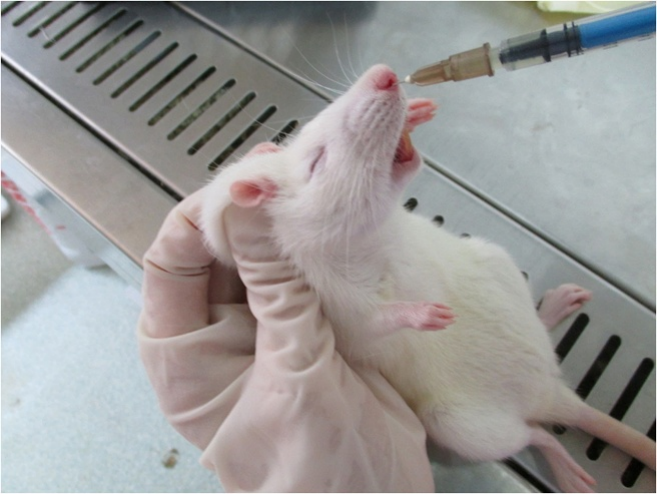
**Dropping** ***A. fumigatus*** **spore suspension into right nasal cavity of rat.** With the rats’ nostrils pointed upward, suspensions of A. fumigatus spores were dropped at a slow speed into the right nasal cavities.

On the second day after the second injection of CPA and CA, rats in experimental groups were anesthetized with intraperitoneal injections of 3% pentobarbital sodium (Sigma, America) at 40 mg/kg. A piece of Merocel sponge was inserted 1.5 cm deep into the right nasal cavity of each experimental rat (Figure 1). Then a suspension of 100 μl of *A. fumigatus* conidia was dropped into the right nasal cavity, with a concentration of 5 × 10^7^ conidia/ml in group A, 1 × 10^7^ conidia/ml in group B, and 1 × 10^6^ conidia/ml in group C (Figure 2). These fungal inoculations were performed for three consecutive days.

### Histopathology analysis

Rats were euthanized with a respiratory-failure dose of 120 mg/kg of pentobarbital sodium (Sigma, America) given by intraperitoneal injection four days after the first administration of *A. fumigatus* conidia. The rats were then decapitated. The skin on the heads was divested and the mandibles were removed. The specimens were soaked in 10% neutral paraformaldehyde for 24 hours. This was followed by decalcification with decalcifying fluid (0.5 mol/L ethylenediaminetetraacetic acid (EDTA), PH 7.4) for one month until soft. The heads were subsequently cut coronally for 2 mm and made into wax blocks, sectioned at 3 μm thickness, and stained with hematoxylin and eosin (HE), Periodic acid-Schiff (PAS), methenamine silver and MUC5B for visualization of the fungi.

Lungs, livers, kidneys and spleens were also extracted for histopathological examination with HE staining.

### Toluidine blue staining

To assist in differentiating MCs from other inflammatory cells, toluidine blue staining was used in this study. Serialized 3 μm-thick sections were deparaffinized, rehydrated, and stained with 0.5% toluidine blue. These sections were stored at 4°C for one night, and subsequently underwent glacial acetic acid differentiation and vitrification by dimethylbenzene. MCs were identified by deep blue staining.

### Immunohistochemical staining for mast cells

Immunohistochemistry was performed using the horseradish peroxidase (HRP) immunohistochemical method. Tissue sections (3 μm) were deparaffinized and rehydrated in distilled water. Endogenous peroxidase activity was blocked by incubation with 0.3% hydrogen peroxide for 10 minutes at room temperature. Antigen retrieval was performed by cooking the sections in citrate solution for 2–3 minutes. The tissue sections were then incubated with anti-mast cell tryptase antibody (AA1IgG1; 1:2000 dilutions) for 35 minutes in a 37°C water bath box after being rinsed once with distilled water and three times with phosphate buffers (PBS) (pH 7.4). The slides were again rinsed with distilled water and PBS (pH 7.4), and exposed to Polymer Helper (direct use type) for 20 minutes in a 37°C water bath box. After washing with distilled water and PBS (pH 7.4) a third time, the slides were exposed to Poly-HRP anti-Mouse IgG (direct use type, no cross reaction with rats) for 20 minutes in a 37°C water bath box. They were then washed a final time in distilled water and PBS (pH 7.4) before diaminobenzidine (DAB) coloration. Lastly, the slides were counterstained with hematoxylin after rinsing with water. MCs were identified by dark-brown staining.

### Statistics

Statistical analyses were performed using SPSS 17.0 (SPSS, Inc., America, English). Comparisons across interventions were evaluated using Chi-square test and repeated measures analysis of variance (REP ANOVA). P-values less than 0.05 were considered to be statistically significant.

## Results

The rats were diagnosed with AIFR if histopathology revealed the presence of *A. fumigatus* hyphae within nasal tissue. During the entire length of the experiment, the Merocel sponges demonstrated no prolapse by remaining in the nostrils of all rats.

### General observations

All experimental rats (groups A, B and C) had decreased appetite after immune inhibitor injection and fungal inoculation, and exhibited lethargy with dulling of their fur. On the second day after the first fungal inoculation, sneezing and nose scratching were observed in each group.

**Figure 3:**
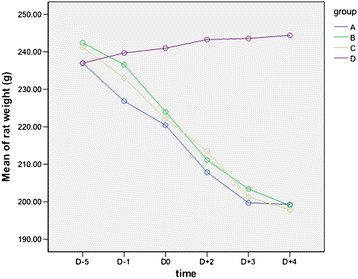
**Changes of rat weight by group (g).** In groups A, B and C, rat weight showed a trend downward after injecting CPA and CA and inoculation of *A. fumigatus*. In group D, rat weight showed a gradually increasing trend during the experiment. (D-5: Five days before the first administration of *A. fumigatus* spores; D-1: One day before the first administration of *A. fumigatus* spores; D0: The first administration day of *A. fumigatus* spores; D + 2: Two days after the first administration of *A. fumigatus* spores; D + 3: Three days after the first administration of *A. fumigatus* spores; D + 4: Four days after the first administration of *A. fumigatus* spores).

Table 3 and Figure 3 illustrate the changes in rat weights throughout the experiment. Groups A, B and C, which were immunosuppressed and inoculated with *A. fumigatus*, had a gradual decline in average weight (P < 0.05), but no significant differences in body weight changes were found between the three groups (P > 0.05). Weights in group D had an increasing trend throughout the entire experiment (P < 0.05).

**Table 3:** **Rat weight changes (g) by group**

Group	n	D-5	D-1	D0	D + 2	D + 3	D + 4
A	10	237.0 ± 14.58	226.9 ± 8.76	220.4 ± 8.90	207.9 ± 19.50	199.7 ± 16.70	199.2 ± 23.11
B	10	242.4 ± 12.60	236.6 ± 11.34	224.0 ± 10.17	211.1 ± 9.82	203.4 ± 7.23	199.0 ± 11.62
C	10	241.3 ± 2.43	233.0 ± 9.26	222.1 ± 8.75	213.6 ± 12.00	201.3 ± 12.50	197.9 ± 14.10
D	10	237.0 ± 9.78	239.7 ± 8.12	241.0 ± 7.75	243.3 ± 6.92	243.6 ± 6.55	244.4 ± 6.43

n: Number of rats; D-5: Five days before the first administration of *A. fumigatus* spores; D-1: One day before the first administration of *A. fumigatus* spores; D0: The first administration day of *A. fumigatus* spores; D + 2: Two days after the first administration of *A. fumigatus* spores;

D + 3: Three days after the first administration of *A. fumigatus* spores; D + 4: Four days after the first administration of *A. fumigatus* spores.

### Absolute neutrophil count

For groups A, B and C, the neutrophil quantities on D0 were less than 0.1 × 10^9^/L, which were lower than the neutrophil counts for group D ((3.7 ± 1.84) × 10^9^/L).

### Histopathological examination

**Figure 4:**
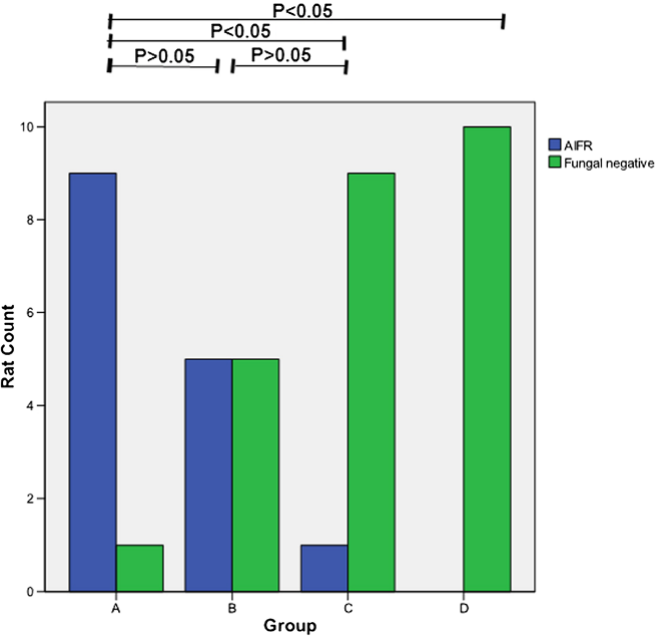
**Rates of acute invasive fungal rhinosinusitis (AIFR) in each group.** Rats in group A had the highest AIFR incidence, followed by rats in groups B and C. No rat in group D was infected by A. fumigatus.

AIFR affected 90% (9/10) of the rats in group A, 50% (5/10) in group B, and 10% (1/10) in group C (Figure 4). The fungal infection rate of group A was significantly higher than that of group B (P > 0.05) and group C (P < 0.05). Only one case of AIFR was established in group C, which had an infection rate that was significantly lower than that of group A (P < 0.05) but not group B (P > 0.05). No AIFR was found in group D.

**Figure 5:**
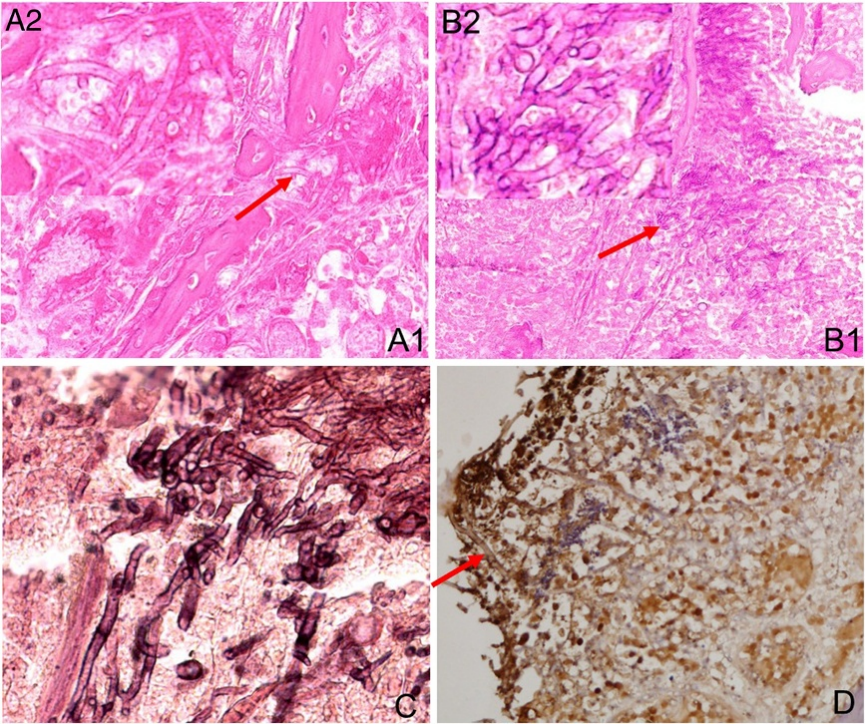
***A. fumigatus*** **hyphae invasion in sinus cavity mucosa of rat with acute invasive fungal rhinosinusitis (AIFR).** Bone resorption is caused by the intrusion of hyphal masses. The hyphae are antler-like. (**A1**. Hematoxylin and eosin staining, ×20; **A2**. Hematoxylin and eosin staining, ×40; **B1**. Periodic acid–Schiff staining, ×10; **B2**. Periodic acid–Schiff staining, ×40; **C**. Methenamine silver staining, ×40; **D**. MUC5B staining, ×40).

**Figure 6:**
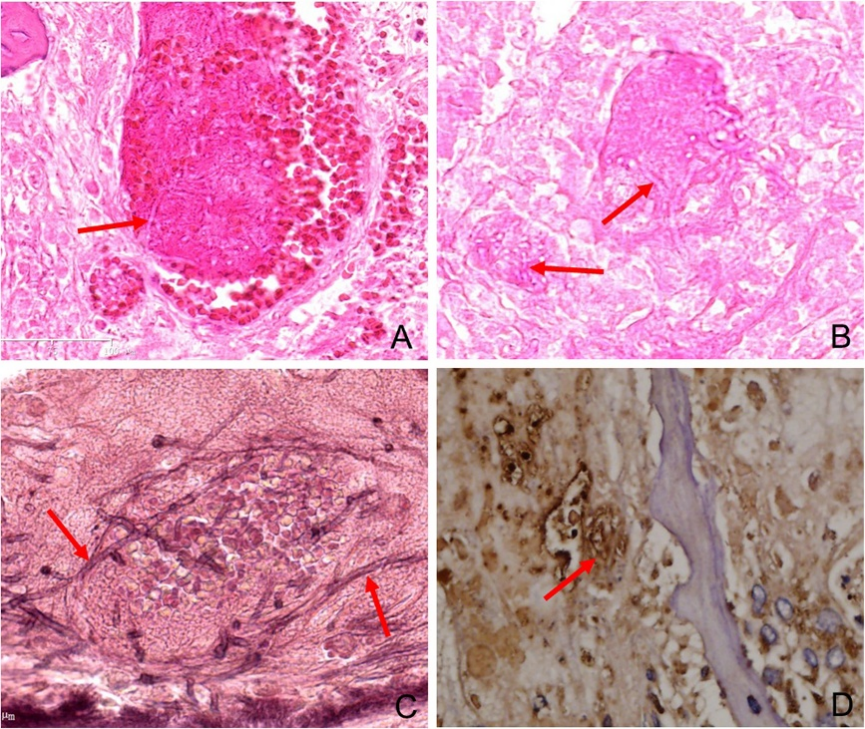
**Vascular invasion of** ***A. fumigatus*** **hyphae in sinus of rat with acute invasive fungal rhinosinusitis (AIFR).** (**A**. Hematoxylin and eosin staining, ×40; **B**. Periodic acid–Schiff staining, ×40; **C**. Methenamine silver staining, ×40; **D**. MUC5B staining, ×40).

**Figure 7:**
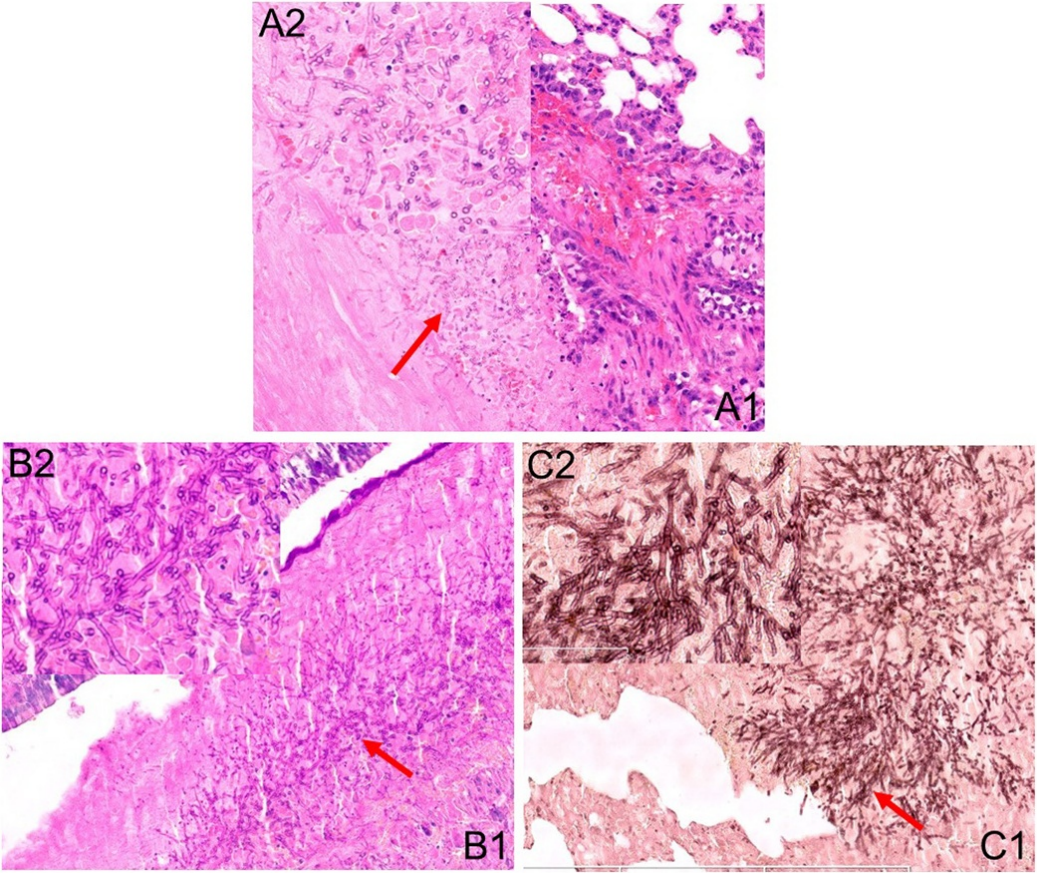
**Abundant** ***A. fumigatus*** **hyphae in lung tissue of group A.** (**A1**. Hematoxylin and eosin staining, ×10; **A2**. Hematoxylin and eosin staining, ×40; **B1**. Periodic acid–Schiff staining, ×10; **B2**. Periodic acid–Schiff staining, ×40; **C1**. Methenamine silver staining, ×10; **C2**. Methenamine silver staining, ×40).

According to our study, the rat nasal septum was the most susceptible site to *A. fumigatus* hyphae, followed by the turbinate, ethmoidal sinus and maxillary sinus (Figure 5). The AIFR rats in group A had a heavy *A. fumigatus* hyphal invasion. The nasal and sinus mucosa contained a large number of fungal components (Figure 5), which caused fungal vasculitis (Figure 6), vascular embolism, hemorrhage, and coagulative necrosis. Simultaneously, bone resorption (caused by the direct intrusion of hyphal masses) was observed without obvious infiltration of inflammatory cells or osteoclast cells near extremely necrotic tissue (Figure 5A, B, C). The hyphae were shaped like antlers, which is a morphological feature of *A. fumigatus*. Large numbers of fungal components and pyogenic granulomas were also seen in the pulmonary tissue of two rats in group A (Figure 7). A lower number of fungal components were found in group B. In addition, only a few invading *A. fumigatus* hyphae were seen in one of the rats belonging to group C. No lung was infected by *A. fumigatus* in groups B or C, and invasion was not found in the brain, liver, kidney or spleen of any group.

Different degrees of epithelial hyperplasia, disorder, and loss, in addition to gland hypertrophy, were found in all the experimental rats. No animal died prior to the euthanization stage.

### Observation of mast cells in AIFR

**Figure 8:**
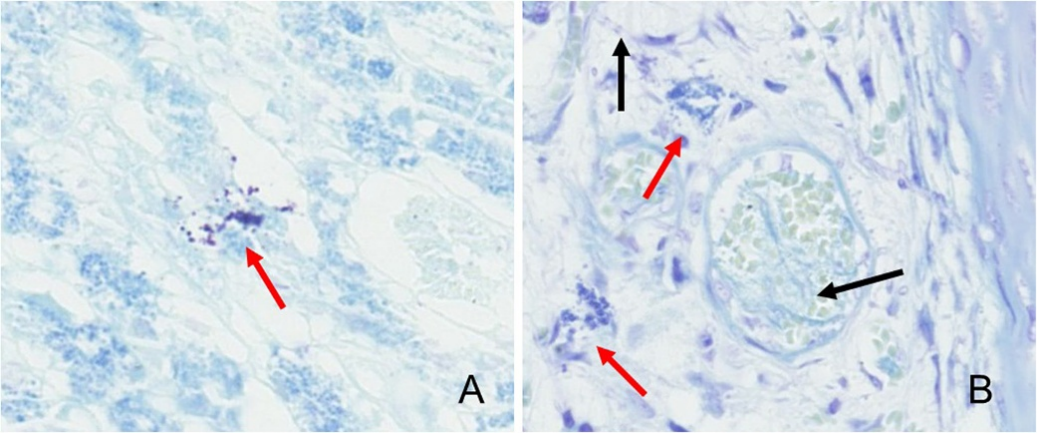
**A. Mast cell (MC) degranulation in hyperplastic mucosa of rat maxillary sinus invaded by** ***A. fumigatus*** **(Toluidine blue staining, ×40). B**. Mast cell (MC) degranulation in rat nasal septum invaded by *A. fumigatus* (Toluidine blue staining, ×40). Red arrow indicates MC degranulation. Black arrow indicates *A. fumigatus* hyphae.

**Figure 9:**
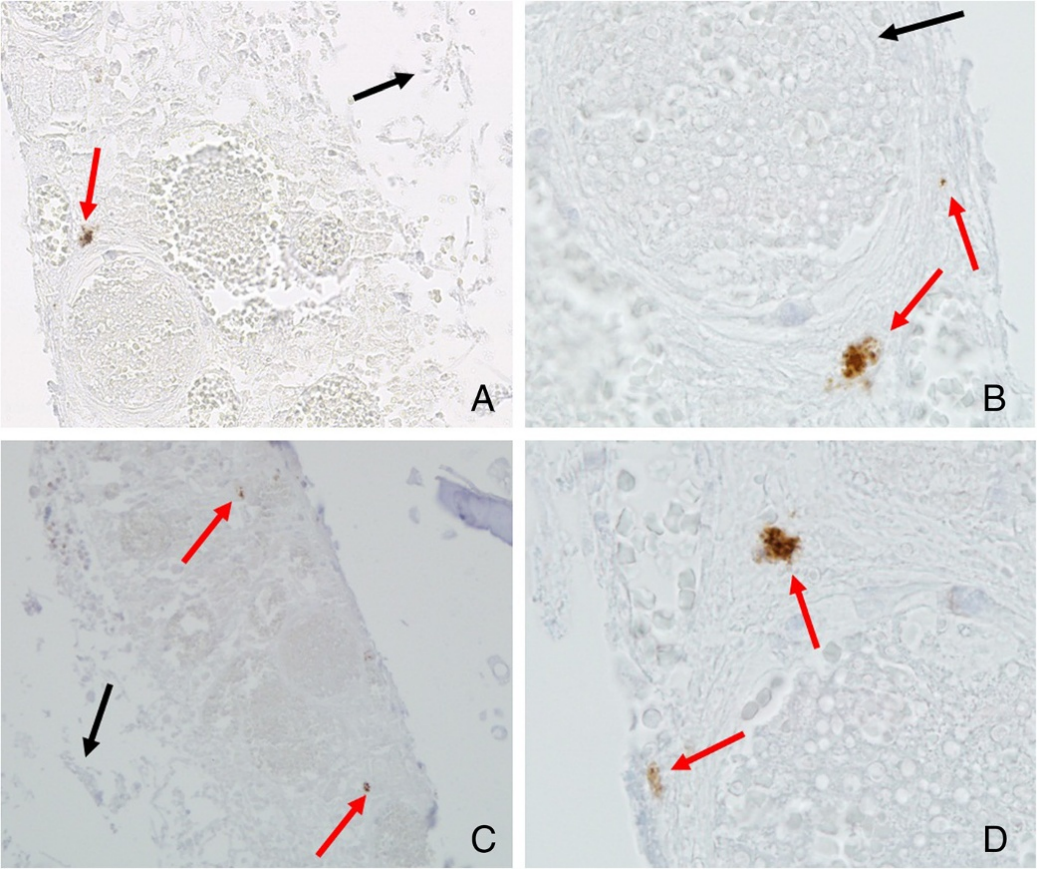
**Tryptase immunohistochemical staining for mast cell (MC) infiltration and degranulation in rat nasal mucosa invaded by** ***A. fumigatus*** **.** Both tryptase-positive MCs and tryptase-positive granules are stained dark brown. (Original magnification × 40 in **A**, ×20 in **C**, and × 100 in **B** and **D**). Red arrow indicates MC infiltration and degranulation. Black arrow indicates *A. fumigatus* hyphae.

**Figure 10:**
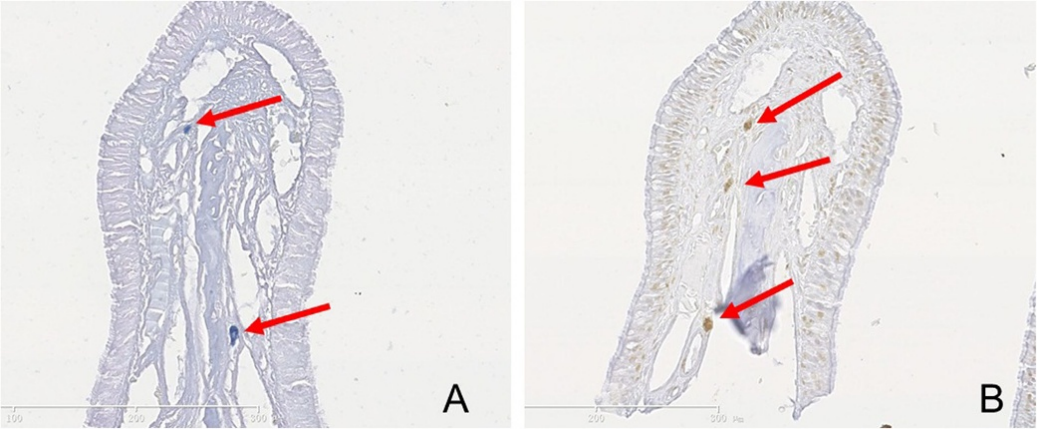
**Mast cell (MC) infiltration in normal rat turbinate of group D.** (**A**. Toluidine blue staining, ×20; **B**. Tryptase immunohistochemical staining for MC infiltration, ×20).

**Figure 11:**
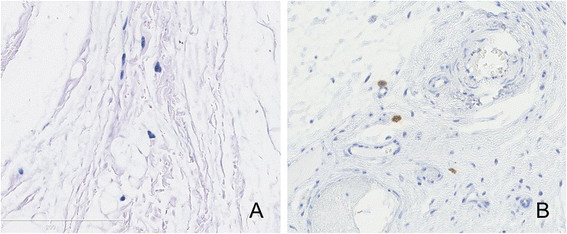
**More mast cell (MC) infiltration in rat nasal peripheral connective tissue in all groups.** No MC degranulation was found. (**A**. Toluidine blue staining, ×20; **B**. Tryptase immunohistochemical staining for MC infiltration, ×20).

MCs were identified by both metachromatic staining with toluidine blue and immunohistochemical staining for tryptase. Using toluidine blue, MCs were identified in tissue sections by their characteristic granular, deep blue-purple metachromatic appearance against blue orthochromatic background tissue (Figure 8). With the tryptase immunohistochemical stain, MCs were identified by dark-brown staining (Figure 9). Compared with the normal nasal cavity (Figure 10), the total number of MCs in *A. fumigatus* invasion areas was not increased. MC degranulation, on the other hand, was only found in or around regions of fungal invasion. While connective tissue around rat noses showed more MC infiltration than nasal cavities, no degranulation was observed in such areas (Figure 11).

## Discussion

We successfully established an AIFR rat model that was stable and easy to operate by utilizing the administration of CPA and CA with subsequent nasal obstruction and fungal inoculation, which allowed us to research MC response to AIFR. This model is based on the development of rat models for bacterial rhinosinusitis and invasive pulmonary aspergillosis [13]-[17],[19],[20],[22],[23]. According to the clinical diagnosis of AIFR, we used the following categories to determine whether the model was successful: general response, immune status, and histopathological examination.

In our study, rats in group A, which were inoculated with 5 × 10^7^ conidia/ml of *A. fumigatus*, had the highest AIFR incidence (90%), followed by rats in group B (50%) and group C (10%). No AIFR occurred in group D. Also, several infiltrating and degranulating MCs were found in regions infected with *A. fumigatus* hyphae. These results indicate that *A. fumigatus* concentration played an important role in the occurrence and development of AIFR, and MCs may have an effect on this disease process.

In our study, we chose SD rats, which were bred with Wistar rats, as the experimental animal, because they are more genetically stable than Wistar. Compared with rabbits and other large animals, rats are less expensive and easier to handle. In addition, they have more blood volume than mice, which is necessary for the repeated sampling of large quantities of blood in this study. Also, based on our pre-experiments, it was difficult to insert Merocel sponges into mouse nasal cavities, and instillation of *A. fumigatus* into mice without previously inserting Merocel sponges caused lung fungal infections more frequently than AIFR.

Because AIFR occurs mainly in immunodeficient patients, we chose to first immunosuppress rats in order to simulate clinical AIFR. Our immunosuppression procedure was based on utilizing CPA in combination with CA. The selection of dosage for CPA and CA was based on a large number of pre-experiments in addition to the results of various articles researching invasive pulmonary aspergillosis in rats [37]-[41]. CPA is an alkylating agent from the oxazaphosphorine group, an immunosuppressant that is widely used in clinical practice [42]. Unfortunately, CPA intake is associated with many serious side effects and toxicity, including mutagenicity, myelosuppression, cardiac toxicity, lung toxicity and urotoxicity, which are mainly mediated by reactive oxygen species and lipid peroxide formation [43],[44]. In our preliminary studies, large doses of CPA induced hematuria in rats, and the mortality rate was high. CA is a type of adrenal cortical hormone with immunosuppressive effects, which can aggravate fungal infections. By adding CA to our immunosuppression procedure, we were able to reduce the dosage of CPA and, thus, rat mortality, which improved the success rate of establishing an AIFR rat model. Several papers have reported that an invasive pulmonary aspergillosis (IPA) rat model can be established under neutropenic conditions (<0.1 × 10^9^/L on the day of fungal inoculation) [37],[38]. In our study, the neutrophil counts on D0 were also less than 0.1 × 10^9^/L. After immunosuppression, all experimental rats demonstrated decreased energy and appetite, in addition to dulling of the fur and weight lost. Under these immunosuppressive conditions, we were able to successfully establish an AIFR rat model with no rat mortality.

In humans, ostiomeatal obstruction is a key precipitating factor in the pathogenesis of sinusitis. Obstructing the ventilation and drainage of the nasal cavity and sinus is one of the major virulence factors of fungal sinusitis. Clinically, the Merocel sponge is often used for patients with epistaxis or after nasal surgery. It has good tissue compatibility and rarely damages the nasal mucosa. Also, it is easy to insert into the nasal cavity and is difficult to prolapse. In 2008, a rabbit model of chronic rhinosinusitis was developed using Merocel sponges, which demonstrated that the Merocel sponge can remain in the nasal cavity for a lengthy period of time [45]. In 2010, Jin et al. reported that bacteria can cause rhinosinusitis under the condition of nasal obstruction with Merocel sponges, while bacteria alone cannot induce rhinosinusitis [15]. Nasal obstruction by Merocel sponges closely mimics the natural course of anthropic invasive fungal rhinosinusitis (IFRS) without surgical damage to local bone or mucosal integrity [24]. These reasons, along with its ease of manipulation, support the use of Merocel sponges in rhinosinusitis studies.

*Aspergillus* is one of the main pathogens found in patients with AIFR [3],[46]. There are almost 900 species of *Aspergillus* in nature, and *A. fumigatus* is the most common one in humans [12]. These organisms are normally found in dust, soil, and the upper respiratory mucosa of healthy individuals. Generally, in healthy individuals, these spores are quickly eliminated by the immune system. However, in immunocompromised patients, inhalation of *A. fumigatus* may lead to AIFR.

Based on a large number of pre-experiments, various concentrations of fungal conidia have different effects on the rat sinus. An *A. fumigatus* concentration of 1 × 10^8^ conidia/ml caused a high mortality rate (>50%). Consequently, we chose 5 × 10^7^, 1 × 10^7^ and 1 × 10^6^ conidia/ml concentrations of *A. fumigatus* as the spore suspensions for this study, and we subsequently compared their different effects on establishing an AIFR rat model.

At present, only two papers reporting on an AIFR animal model have been published. In 2007, Rodriguez et al. developed a mouse model of AIFR through depleting neutrophils using anti-Gr-1 monoclonal antibody combined with the intranasal administration of *A. fumigatus* [5]. In this experiment, they failed to successfully utilize CPA in developing their animal model, which may be related to an inappropriate dose of cyclophosphamide. In contrast to this study, we immunosuppressed experimental rats by using CPA and CA, which are easily obtainable, commonly used immune inhibitors in the clinic. In 2013, a group of researchers established a rat model of AIFR [24]. They obstructed the rat nasal cavity with Merocel sponges for 5 days, and then injected CPA for immunosuppression, followed by inoculating *A. fumigatus* for a week. The entire experiment lasted for 18 days. In contrast, we developed a novel method to establish an AIFR rat model that better simulates the disease process in humans. In order to mimic the clinical process of AIFR formation, we injected rats with immune inhibitors first, and then inserted Merocel sponges into the rats’ nasal cavities, followed by inoculating *A. fumigatus* for three days. The entire experimental process lasted for only nine days. Compared with the two published studies, our methods are easier to operate and provided a stable fungal infection rate.

MCs, which play key roles in innate and adaptive immunity, are important effector cells in protective immune responses against pathogens [28]-[32]. While increasingly more data indicate that MCs play a crucial role in host defense, especially against bacteria [29], very little is known about the relevance of MC effects in fungal infections. In addition, no study has reported on the role of MCs in AIFR. Here, we observed the changes of MCs in AIFR rats. Toluidine blue, which is reliable in identifying MCs, and immunohistochemical staining for tryptase, which is a highly sensitive and specific method for MC identification [47], were used in this study. We observed that the total number of MCs in *A. fumigatus* invasion areas were not increased compared to normal rat nasal cavities. Even with low quantities, MC degranulation was found only in regions of *A. fumigatus* invasion or nearby perivascular areas. This indicates that the main role of MCs may not consist of direct phagocytosis after migration to fungal infection zones in AIFR, but instead may involve degranulation or the release of cytokines and chemokines involved in innate and adaptive immunity. Several researchers have reported that the functional status of MCs is represented by MC degranulation, and the ability of MCs to degranulate in response to various stimuli is the very basis for their biological activity [48]. Through degranulation, MCs release several classes of preformed mediators, including histamine and proteases, contributing to host defense. In addition, MCs also employ numerous de novo synthesized mediators, many of which have been shown to be involved in protective host responses to fungal pathogens, such as IFN-γ [49].

## Conclusions

Various fungal concentrations have different effects on establishing an AIFR rat model. The optimal fungal concentration plays an important role in the course of formation and development of AIFR. By inoculating a suspension of 5 × 10^7^ conidia/ml, a stable AIFR rat model that is easy to operate can be established successfully. The MC data of the present study indicate that MCs may mainly play a role through degranulation or the release of cytokines and chemokines involved in innate and adaptive immunity, instead of direct phagocytosis of fungi in AIFR. The immunoregulatory role of MCs in both protective and destructive AIFR immune responses should be further evaluated, and detailed studies of MC function will enable the development of more effective therapies for AIFR via MC manipulation. Our AIFR rat model can be used to study the nasal immune mechanisms against *A. fumigatus*, and may be useful for the research of AIFR and MCs in the future.

## Authors’ original submitted files for images

Below are the links to the authors’ original submitted files for images. Authors’ original file for figure 1 Authors’ original file for figure 2 Authors’ original file for figure 3 Authors’ original file for figure 4 Authors’ original file for figure 5 Authors’ original file for figure 6 Authors’ original file for figure 7 Authors’ original file for figure 8 Authors’ original file for figure 9 Authors’ original file for figure 10 Authors’ original file for figure 11 Authors’ original file for figure 12 Authors’ original file for figure 13
