# Supplementary figures and images for: A novel fungus concentration-dependent rat model for acute invasive fungal rhinosinusitis: an experimental study
Source: BMC Infect Dis. 2014 Dec 20;14:3856. doi: 10.1186/s12879-014-0713-y (PMC4297382; doi:10.1186/s12879-014-0713-y)

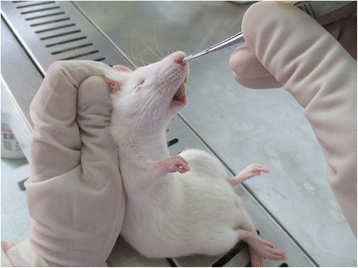

Supplement: Supplementary file 1 — Authors’ original file for figure 1 [file 12879_2014_713_MOESM1_ESM.gif]

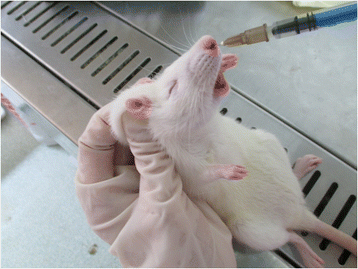

Supplement: Supplementary file 2 — Authors’ original file for figure 2 [file 12879_2014_713_MOESM2_ESM.gif]

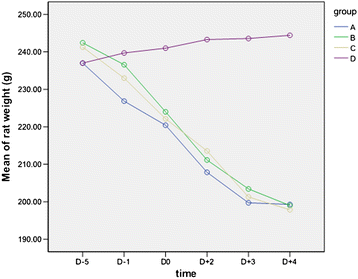

Supplement: Supplementary file 3 — Authors’ original file for figure 3 [file 12879_2014_713_MOESM3_ESM.gif]

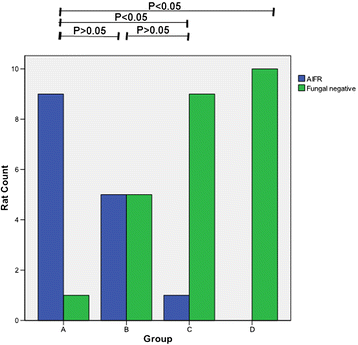

Supplement: Supplementary file 4 — Authors’ original file for figure 4 [file 12879_2014_713_MOESM4_ESM.gif]

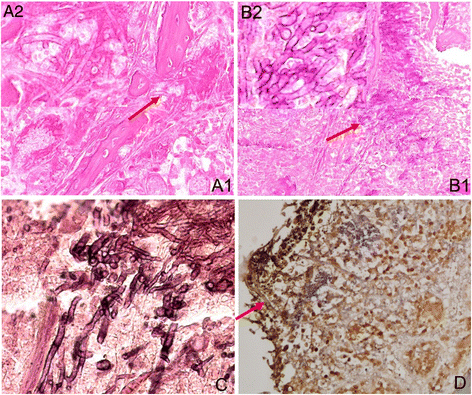

Supplement: Supplementary file 5 — Authors’ original file for figure 5 [file 12879_2014_713_MOESM5_ESM.gif]

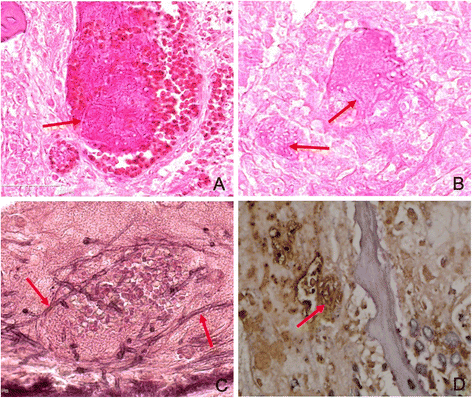

Supplement: Supplementary file 6 — Authors’ original file for figure 6 [file 12879_2014_713_MOESM6_ESM.gif]

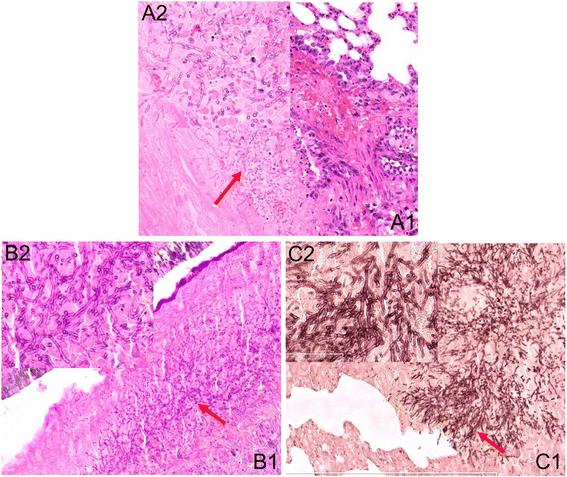

Supplement: Supplementary file 7 — Authors’ original file for figure 7 [file 12879_2014_713_MOESM7_ESM.gif]

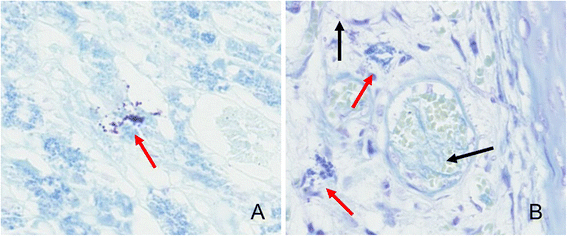

Supplement: Supplementary file 8 — Authors’ original file for figure 8 [file 12879_2014_713_MOESM8_ESM.gif]

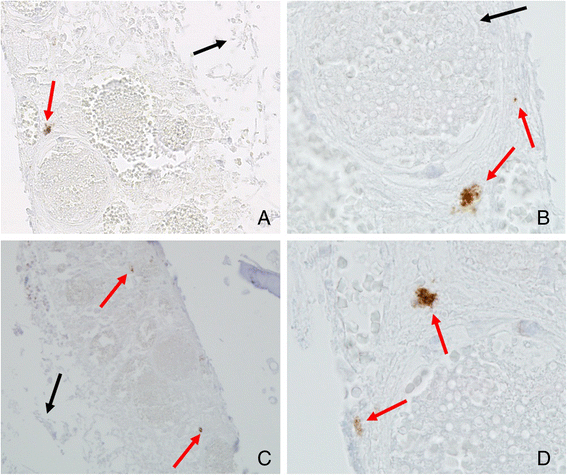

Supplement: Supplementary file 9 — Authors’ original file for figure 9 [file 12879_2014_713_MOESM9_ESM.gif]

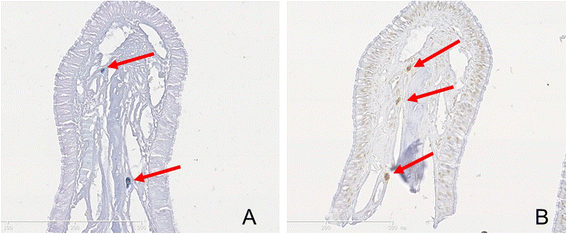

Supplement: Supplementary file 10 — Authors’ original file for figure 10 [file 12879_2014_713_MOESM10_ESM.gif]

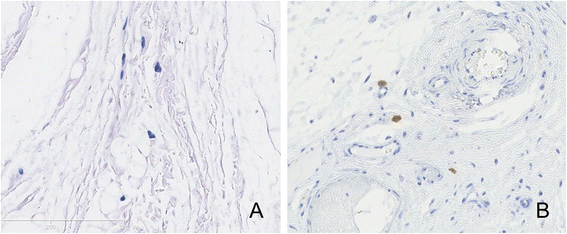

Supplement: Supplementary file 11 — Authors’ original file for figure 11 [file 12879_2014_713_MOESM11_ESM.gif]

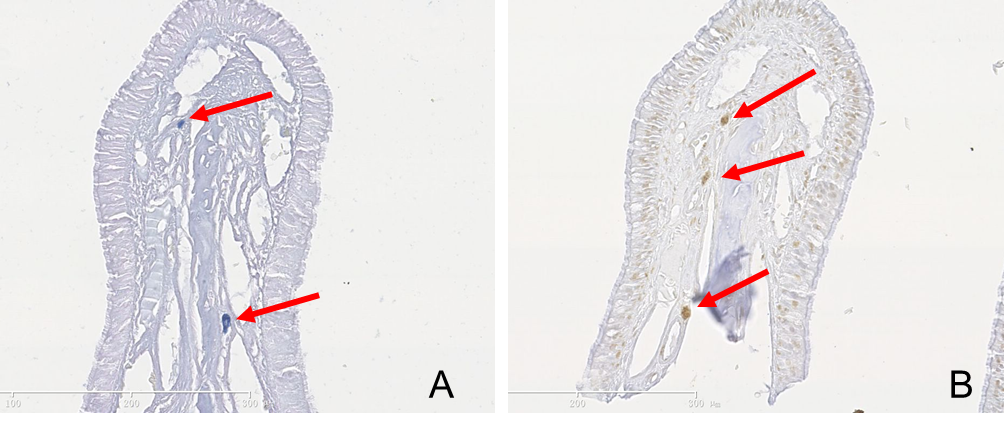

Supplement: Supplementary file 12 — Authors’ original file for figure 12 [file 12879_2014_713_MOESM12_ESM.tiff]

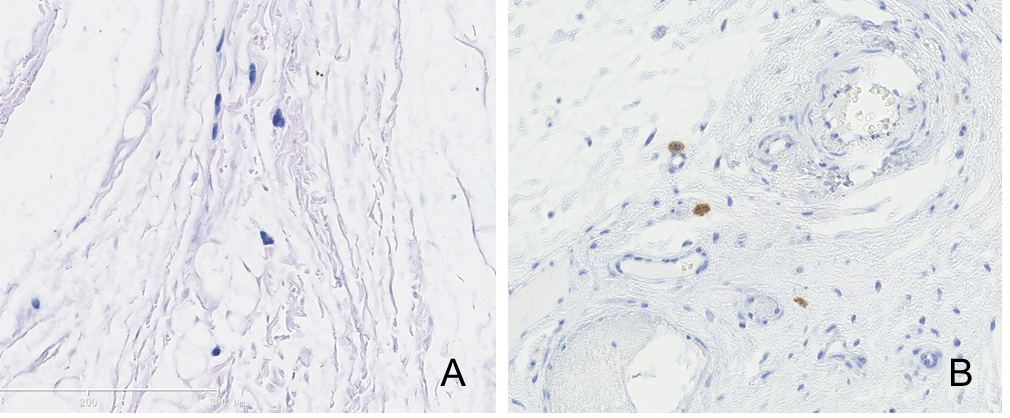

Supplement: Supplementary file 13 — Authors’ original file for figure 13 [file 12879_2014_713_MOESM13_ESM.tiff]
